# The Genetic Diversity of *Pleurozium schreberi*: A Preliminary Study Based on the atpB-rbcL

**DOI:** 10.3390/plants12193487

**Published:** 2023-10-05

**Authors:** Paweł Świsłowski, Paweł J. Domagała, Małgorzata Rajfur

**Affiliations:** Institute of Biology, University of Opole, Oleska St. 22, 45-052 Opole, Poland; pdomagala@uni.opole.pl (P.J.D.); rajfur@uni.opole.pl (M.R.)

**Keywords:** genetics, moss, sequences, chloroplast DNA, haplotypes

## Abstract

*Pleurozium schreberi* is a common and widespread species that has been the object of many studies, and its biology and ecology are well known. However, genetic studies on this species are limited or even absent. Because of the lack of any data about the genetic diversity of the moss species *P. schreberi* in Poland, the present paper describes the results of the studies carrying out for the first time this kind of research based on the atpB-rbcL spacer sequences of chloroplast DNA. A total of 35 specimens of *P. schreberi* from 19 locations in Poland were sampled. Total genomic DNA was extracted, amplified, and sequenced, and all obtained sequences were analyzed. Our findings suggest the low genetic diversity of *P. schreberi* in Poland. We detected four different haplotypes, shared between different populations.

## 1. Introduction

*Pleurozium schreberi* (Willd. ex Brid.) Mitt. is widespread in the temperate zone of the Northern Hemisphere [1,2]; however, some records from tropical zones are present, i.e., South America [3] and Africa [4]. It can be found on soil on the floor of coniferous and mixed forest, occasionally on rotten wood, in grassland at forest margins, and in heathland [5]. In Poland, *P. schreberi* is widespread in pine forests (*Leucobryo*-*Pinetum* and *Peucedano*-*Pinetum*), which are the dominant type of forest in Poland [6]. *Leucobryo*-*Pinetum* pine forests are widespread in western, southern and central Poland, while *Peucedano*-*Pinetum* forests occur in the north-western area of Poland. In both cases, the tree stand consists mainly of the Scots pine (*Pinus sylvestris*) and a small share of silver birch (*Betula pendula*), as well as the European spruce (*Picea abies*) in the *Peucedano*-*Pinetum* forests. These forests have been and are used economically; in many areas, the tree stand is homogeneous (monotypization) and consists of Scots pine (*P. sylvestris*) only [7].

This species was and is the object of many studies, i.e., [8,9,10,11]. In the literature, we can find studies of the growth and reproduction of this species [12,13], the influence of various factors on its acclimatization [8,14,15,16], and biomonitoring studies [17,18,19,20,21]. There are little-known medical and ethnopharmacological reports on these particular usages for this species, and so further assessment is required in this area of study [22]. The genetic studies on this species are limited or even absent [23,24]. Because of the lack of any data about the genetic structure of *P. schreberi* in Poland, we decided to carry out for the first time this type of research based on the atpB-rbcL spacer sequences of chloroplast DNA.

We attempted to verify the research hypothesis that mosses are characterized by low genetic diversity due to the homogeneous type of habitat they populate.

## 2. Results and Discussion

We successfully obtained 35 atpB-rbcL spacer sequences of 547 bp in length. The obtained results show very low differences among analyzed sequences. A total of four different haplotypes were identified, with a haplotype diversity (Hd) of 0.4672. The number of variable sites, as well as the number of polymorphic (segregating) sites, was only two (see Table 1). The total number of mutations in analyzed sequences was two. The obtained average number of nucleotide differences (k) was 0.5714, and the nucleotide diversity (Pi) was 0.00104.

**Figure 1:**
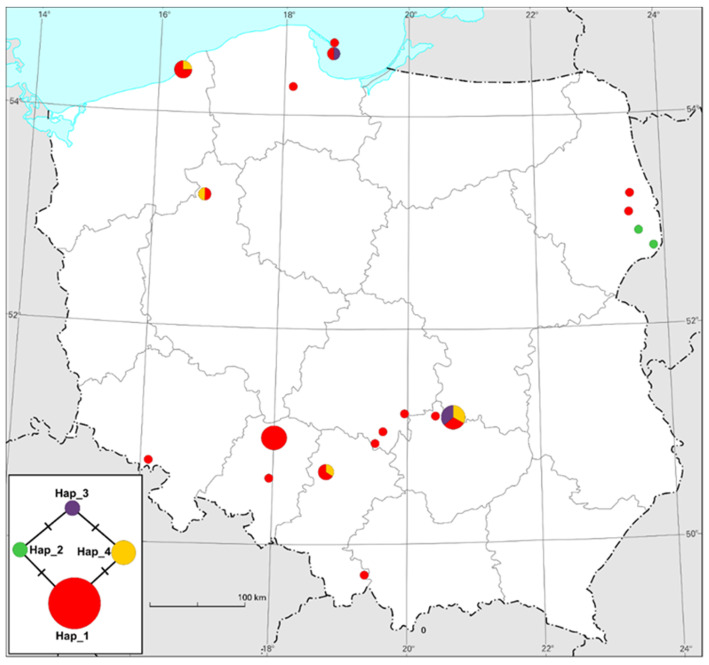
The locations of the examined specimens, their relation to obtained haplotypes and the median-joining haplotype network for atpB-rbcL spacer sequences. Different colors correspond to specific haplotypes (Hap_1—red; Hap_2—green; Hap_3—violet; Hap_4—yellow) and the circle size is proportionate to the number of analyzed individuals. Hatch marks along the edges represent the number of mutations between nodes.

The most common haplotype (Hap_1) is present in 25 specimens from 16 widespread localities around Poland (Figure 1). The additional sequence MK313952.1, obtained from NCBI GenBank, also shares Hap_1 (see Table 2). It seems that this haplotype may be more widely distributed in Europe, or even throughout its range. Unfortunately, due to the lack of data, we are unable to confirm this hypothesis at this time. The haplotypes Hap_3 and Hap_4 are much less common, but also widespread. Of particular interest is haplotype Hap_2, which is restricted to two specimens from Białowieża Forest and Ladzka Forest (NW part of Białowieża Forest). A generated haplotype network showed that each obtained haplotype differs from the others by only one mutation (Figure 1).

It seems that such low sequence variation in the atpB-rbcL spacer is common in bryophytes. Studies of distributed mosses *Pyrrhobryum mnioides* and *Ceratodon purpureus* also show very low diversity in the same cpDNA region [25,26]. In the case of *P. mnioides*, nine haplotypes were detected and sequences of 559 bp consist of 4.9% variable sites. Research carried out on the microsatellite markers of many species of bryophytes, i.e., *Isothecium myosuroides* [27], *Leptodon smithii* [28], *Polytrichum commune* [29], and *Trichocolea tomentella* [30], showed that populations occupying disturbed habitats have a low level of genetic diversity. However, our samples were collected away from any anthropogenic activity, but mainly in managed pine forests. In the past, errors in forest management in Poland, such as fertilization, grazing, the influence of air and surface waters polluted with nitrogen compounds, overexposure of trees, and rapid mineralization of the forest litter, caused excessive development of grasses (cespitization) and some species of moss (bryophytization), e.g., *P. schreberi* [7]. It cannot be ruled out that bryophytization contributes to the rapid spread of some haplotypes over a given area.

Of course, microsatellite markers are much more reliable in this kind of analysis than atpB-rbcL spacer sequences; however, a similar correlation cannot be ruled out.

On the other hand, the sequence variation of atpB-rbcL in Poland could be higher than detected. Certainly, we have only found four haplotypes. However, it should be noted that samples from the same locality or area have different haplotypes. For example, specimens from Darłowo, Lubliniec, Piła, and Stary Janów belong to haplotypes Hap_1 and Hap_4. We collected between two and four samples at these locations (see Table 3). In our opinion, collecting and analyzing several dozen samples from a given locality would probably allow us to detect other haplotypes. We are aware that the obtained results may not be reliable because of the small number of samples from each locality (see Table 3).

The obtained nucleotide composition was A—43.9%, G—9.99%, C—7.86%, T—38.2%. The adenine and thymine content is similar to that of other bryophytes, i.e., *Campylopus* (A—40.7%), *Rhytidium, Hedwigia*, and *Thuidium* (T—44.5%). The rich A + T content is typical for noncoding spacers due to low functional constraints [31].

### Limitations of the Study

The findings of this study have to be seen in light of some limitations. The first limitation is the restriction on sampling mosses in Poland for scientific research purposes. Polish law regulations allow the collection of 6 moss species (under partial protection), which can be used for research. Obtaining permits for collecting samples from the Regional Directorate for Environmental Protection limits our research opportunities. Therefore, the authors limited themselves to collecting samples mainly from the Opolskie Voivodeship (place of work). The remaining locations were random, and related to the authors’ other research. Another limitation was the restricted funding for research. The obtained funds allowed us to analyze only one fragment of cpDNA (atpB-rbcL), which is used in phylogenetic analyses for plants, including mosses.

In conclusion, the authors are aware of the limitations contained in this short communication. These are as follows: The number of collected samples was limited, the samples were collected in a low number of locations, and, because of limited funding, the research was carried out on one cpDNA marker only.

## 3. Materials and Methods

### 3.1. Specimen Sampling

A total of 35 specimens of *P. schreberi* were collected from 19 locations for this study (June–September 2021), on bright days and in the afternoons, according to their photosynthesis [32]. The locations were random, and related to the authors’ other research. Samples were collected during field expeditions associated with other experiments, and, on occasion, also in places where this species of moss was found and identified. Therefore, the authors limited themselves to collecting samples mainly from the Opolskie Voivodeship (place of work). The locations of the examined specimens are shown in Table 3. This species has loosely pinnate branches and a bright red stem showing through translucent green leaves, and is usually easy to recognize. When dry, the red stems only become visible on wetting, or after scraping leaves off the stem. Shoots are several centimeters long [33]. The locations of the examined specimens are shown in Figure 1 and provided in Table 3. *P. schreberi* avoids calcareous or base-rich habitats, and is most commonly found amongst grass and heather on heathland and in open heath forests. *P. schreberi* also commonly occurs in bogs with ling heather (*Calluna*) and cotton-grass (*Eriophorum vaginatum*) [33]. The mosses were collected in accordance with the current law and the guidelines set out in the Environment Ministry’s regulation (hand collection, no less than 75% of each sod was kept) [34]. After obtaining permission from the Regional Directorate for Environmental Protection, the mosses were identified and collected by the corresponding author. Mosses were sampled according to the guidelines of the ICP Vegetation guide for biomonitoring studies. Samples were collected away from tree canopy cover, roads, or any anthropogenic activity [35]. After sampling, the specimens were stored in paper bags until DNA extraction.

### 3.2. DNA Isolation

Leaves of sampled specimens were frozen in liquid nitrogen and homogenized in a mortar to obtain a powder. Total genomic DNA was extracted from the powdered tissue using a Genomic Mini AX Plant kit (A&A Biotechnology, Gdańsk, Poland), according to the manufacturer’s protocol.

The PCR amplifications were performed in a volume of 50 μL, using ready-to-use mix—PCR Mix Plus (A&A Biotechnology)—and primer pairs. Universal primer pair rbcL-1 (5′-AACACCAGCTTTRAATCCAA-3′) and atpB-1 (5′-ACATCKARTACKGGACCAATAA-3′), proposed by Chiang and co-authors, were used in this study [36]. The atpB-rbcL spacer is one of the most frequently used plastid spacers among bryophytes [37]. According to Stech and Quandt [37], the atpB-rbcL spacer provides more information in terms of informative sites than the other commonly used plastid spacers like trnL-F, psbA-trnH, and rps4-trnS.

The PCR profile consists of 3 min initial denaturation at 94 °C, followed by 30 cycles of 40 sec denaturation at 94 °C, 50 sec annealing at 50 °C, and 80 sec elongation at 72 °C. The terminal elongation was 7 min 72 °C.

The sequencing was performed by A&A Biotechnology, Gdynia, Poland. All the obtained sequences were checked using the BLAST online tool (https://blast.ncbi.nlm.nih.gov accessed on 6 October 2022) to verify their correctness. The sequences were deposited in GenBank under accession numbers OP860515 to OP860549 [38].

### 3.3. DNA Analysis

All obtained sequences were analyzed in FinchTV (Geospiza.com, accessed on 6 October 2022) and aligned using ClustalW implemented in MEGA X software [39]. To avoid the influence of missing data, the ends of the sequences were trimmed. The standard genetic indices, i.e., nucleotide composition, nucleotide diversity (ND), number of haplotypes (H), haplotype diversity (hd), number of segregating sites (S), number of variable sites (V), total number of nucleotide differences (TM), and average number of nucleotide differences (k) were calculated using DnaSP v.6 [40]. The haplotype networks for each gene were constructed using a Median-Joining Network method [41] in PopART v.1.7 software [42].

The atpB-rbcL spacer sequence of *Pleurozium schreberi* obtained from NCBI GenBank was attached to the analysis. The accession number for this additional sequence is MK313952. The specimen originates from Zliv in the Czech Republic.

The pairwise distances for each obtained haplotype and additional sequence MK313952.1 were calculated in MEGA X software using the number of differences model [39].

## 4. Conclusions

Our preliminary study showed a low genetic variation in atpB-rbcL spacer sequences among *P. schreberi* specimens in some areas of Poland. One of the hypothetical reasons for this result could be that we collected mosses in their homogeneous habitat. Our samples were collected in pine forests (*Leucobryo*-*Pinetum* and *Peucedano*-*Pinetum*), which are the typical habitat of this species in Poland. Most of these pine forests have been and are used economically, which may also affect the obtained results.

The research should be repeated for other combinations of cpDNA markers, i.e., psbA-trnH, trnL-F, and rps4-trnS, with more samples from each location. In the future, it is worth considering these markers, as well as microsatellite markers or NGS technology, to investigate the genetic diversity of mosses, not only in Poland, but also throughout Europe and the world.

## Figures and Tables

**Table 1:** Polymorphic sites of analyzed sequences of the atpB-rbcL spacer of *Pleurozium schreberi*.

Haplotype/Sequence	Position in the Alignment	
	79	475
Hap_1	G	G
Hap_2	G	A
Hap_3	A	A
Hap_4	A	G
MK313952.1	G	G

**Table 2:** Number of nucleotide differences among haplotypes and additional sequence MK313952.1 (Zliv, Czech Republic).

Haplotype/ Sequence	Hap_1	Hap_2	Hap_3	Hap_4	MK313952.1
Hap_1					
Hap_2	1				
Hap_3	2	1			
Hap_4	1	2	1		
MK313952.1	**0**	1	2	1	

**Table 3:** Sampling sites of mosses.

No.	Locality	GPS	Haplotype
1.	Stobrawa Landscape Park (Opole Voivodeship)	N 50°53′01″ E 17°50′38″	Hap_1
2.		N 50°53′19″ E 17°52′46″	Hap_1
3.		N 50°53′32″ E 17°51′41″	Hap_1
4.		N 50°53′25″ E 17°52′14″	Hap_1
5.		N 50°53′22″ E 17°52′02″	Hap_1
6.		N 50°53′22″ E 17°51′43″	Hap_1
7.	Bialowieza Forest; Grudki near Białowieża (Podlaskie Voivodeship)	N 52°40′37″ E 23°49′44″	Hap_2
8.	Knyszyńska Forest: Międzyrzecze (Podlaskie Voivodeship)	N 53°15′53″ E 23°28′10″	Hap_1
9.	Paczyn near Kamienna Góra (Lower Silesian Voivodeship)	N 50°44′24″ E 15°54′10″	Hap_1
10.	Madeje near Żywiec (Silesian Voivodeship)	N 49°40′26″ E 19°20′38″	Hap_1
11.	Ladzka Forest (NW part of Białowieża Forest) (Podlaskie Voivodeship)	N 52°52′47″ E 23°37′29″	Hap_2
12.	Knyszyńska Forest (Podlaskie Voivodeship)	N 53°04′23″ E 23°29′30″	Hap_1
13.	Hel (Pomeranian Voivodeship)	N 54°39′05″ E 18°45′19″	Hap_3
14.	Jurata (Pomeranian Voivodeship)	N 54°40′29″ E 18°43′32″	Hap_1
15.	Hel (Pomeranian Voivodeship)	N 54°37′09″ E 18°48′01″	Hap_1
16.	Piła (env.) (Greater Poland Voivodeship)	N 53°10′27″ E 16°46′55″	Hap_4
17.	Piła (env.) Greater Poland Voivodeship	N 53°10′28″ E 16°46′48″	Hap_1
18.	Darłowo (env.) (West Pomeranian Voivodeship)	N 54°24′58″ E 16°27′52″	Hap_1
19.		N 54°24′57″ E 16°27′54″	Hap_1
20.		N 54°24′59″ E 16°27′56″	Hap_1
21.		N 54°25′02″ E 16°28′03″	Hap_4
22.	Lubliniec (env.) (Silesian Voivodeship)	N 50°37′27″ E 18°40′57″	Hap_4
23.		N 50°37′27″ E 18°40′55″	Hap_1
24.		N 50°37′27″ E 18°40′55″	Hap_1
25.	Stary Janów near Stąporków (Świętokrzyskie Voivodeship)	N 51°08′10″ E 20°29′11′′	Hap_4
26.	Stary Janów near Stąporków (Świętokrzyskie Voivodeship)	N 51°08′15″ E 20°29′52″	Hap_1
27.	Wąsosz (Świętokrzyskie Voivodeship)	N 51°08′23″ E 20°28′22″	Hap_4
28.	Końskie (Świętokrzyskie Voivodeship)	N 51°10′17″ E 20°25′43″	Hap_3
29.	Forest between Dęba and Nowy Kazanów (Świętokrzyskie Voivodeship)	N 51°10′31″ E 20°18′11″	Hap_4
30.	Wieżyca (Pomeranian Voivodeship)	N 54°13.56″ E 18°07′43″	Hap_1
31.	Zielonka near Radomsko (Łódź Voivodeship)	N 50°58′39″ E 19°24′54′′	Hap_1
32.	Radomsko (Łódź Voivodeship)	N 51°02′09″ E 19°26′30′′	Hap_1
33.	Przedbórz (Łódź Voivodeship)	N 51°05′18″ E 19°53′54′′	Hap_1
34.	Stąporków (Świętokrzyskie Voivodeship)	N 51°07′37″ E 20°33′24′′	Hap_1
35.	Prószków (Opole Voivodeship)	N 50°35′09″ E 17°48′49′′	Hap_1

## Data Availability

Sequences used in this study were deposited in NCBI GenBank under accession numbers: OP860515-OP860549.
